# Supplementary material for: Long-Term Administration of Antioxidant N-Acetyl-L-Cysteine Impacts Beta Cell Oxidative Stress, Insulin Secretion, and Intracellular Signaling Pathways in Aging Mice
Source: Antioxidants (Basel). 2025 Mar 31;14(4):417. doi: 10.3390/antiox14040417 (PMC12023964; doi:10.3390/antiox14040417)
Supplement: Supplementary file 1 [file antioxidants-14-00417-s001.zip › antioxidants-3469153-supplementary.pdf]

Supplemental Table S1. Antibodies for Immunohistochemistry/immunofluorescence and western blot analyses.

Primary Antibody	Dilution	Company
Mouse Anti-8OHdG	1:50*	Santa Cruz Biotechnology, Dallas, TX, USA (sc66036)
Rabbit Anti-AKT	1:3000[W]	Cell Signaling Technology, Danvers, MA, USA (#9727)
Rabbit Anti-pAKT (Ser473)	1:2000[W]	Cell Signaling Technology, Danvers, MA, USA (#4060)
Rabbit Anti-Cleaved Caspase 3	1:1000[W]	Cell Signaling Technology, Danvers, MA, USA (#9661)
Rabbit Anti-Caspase 3	1:1000[W]	Cell Signaling Technology, Danvers, MA, USA (#9662)
Rabbit Anti-eIF2α	1:1000[W]	Cell Signaling Technology, Danvers, MA, USA (#9722)
Rabbit Anti-peIF2α (Ser51)	1:1000[W]	Cell Signaling Technology, Danvers, MA, USA (#3597)
Rabbit Anti-ERK1/2	1:3000[W]	Cell Signaling Technology, Danvers, MA, USA (#9102)
Rabbit Anti-pERK1/2 (Thr202/Ty204)	1:3000[W]	Cell Signaling Technology, Danvers, MA, USA (#9101)
Rabbit Anti-FoxO1	1:1000[W]	Cell Signaling Technology, Danvers, MA, USA (#2880)
Mouse Anti-Glut2	1:50/1:1000[W]	Santa Cruz Biotechnology, Dallas, TX, USA (sc518022)
Rabbit Anti-pIGFIR (Tyr1131)/p-IRβ (Tyr1146)	1:1000[W]	Cell Signaling Technology, Danvers, MA, USA (#3021)
Rabbit Anti-Insulin	1:200*	Cell Signaling Technology, Danvers, MA, USA (C27C9)
Mouse Anti-Insulin	1:800	Sigma, Saint Louis, MO, USA (I2018)
Mouse Anti-IRβ	1:1000[W]	Santa Cruz Biotechnology, Dallas, TX, USA (sc57342)
Rabbit Anti-pIRS-1 (Ser612)	1:1000[W]	Cell Signaling Technology, Danvers, MA, USA (#3203)
Rabbit Anti-Munc18-1A	1:2000[W]	Abcam Inc, Cambridge, MA, USA (ab3451)
Rabbit Anti-NFκB	1:1000[W]	Cell Signaling Technology, Danvers, MA, USA (#8242)
Rabbit Anti-pNFκB (Ser536)	1:1000[W]	Cell Signaling Technology, Danvers, MA, USA (#3033)
Rabbit Anti-PARP	1:1000[W]	Cell Signaling Technology, Danvers, MA, USA (#9532)
Mouse Anti-PCNA	1:1000[W]	Santa Cruz Biotechnology, Dallas, TX, USA (sc56)
Guinea Pig Anti-PDX1	1:800*	Gift from Dr. C. Wright University of Vanderbilt, USA
Rabbit Anti-PPARγ	1:1000[W]	Cell Signaling Technology, Danvers, MA, USA (#2435)
Mouse Anti-αSMA	1:200[+]	Abcam Inc, Cambridge, MA, USA (ab7817)
Mouse Anti-Smad2/3	1:250[W]	Santa Cruz Biotechnology, Dallas, TX, USA (sc133098)
Mouse Anti-SNAP25	1:50/1:1000[W]	Santa Cruz Biotechnology, Dallas, TX, USA (sc20038)
Mouse Anti-Syntaxin 1A	1:50*/1:1000[W]	Santa Cruz Biotechnology, Dallas, TX, USA (sc12736)
Mouse Anti-TNFα	1:250[W]	Santa Cruz Biotechnology, Dallas, TX, USA (sc52746)
Rabbit Anti-VAMP2	1:1000[W]	Abcam Inc, Cambridge, MA, USA (ab181869)

Secondary Antibody	Dilution	Company
Goat Anti-Mouse	1:50	Jackson Immunoresearch, West Grove, Pam USA
Goat Anti-Rabbit	1:50	Jackson Immunoresearch, West Grove, Pam USA
Anti-Mouse HRP-linked	1:2000-3000	Cell Signaling Technology, Danvers, MA, USA
Anti-Rabbit HRP-linked	1:2000-3000	Cell Signaling Technology, Danvers, MA, USA

*Citrate Antigen Retrieval (pH 6.0) used; [+]0.2% Triton used; [W]Used in western blot probing

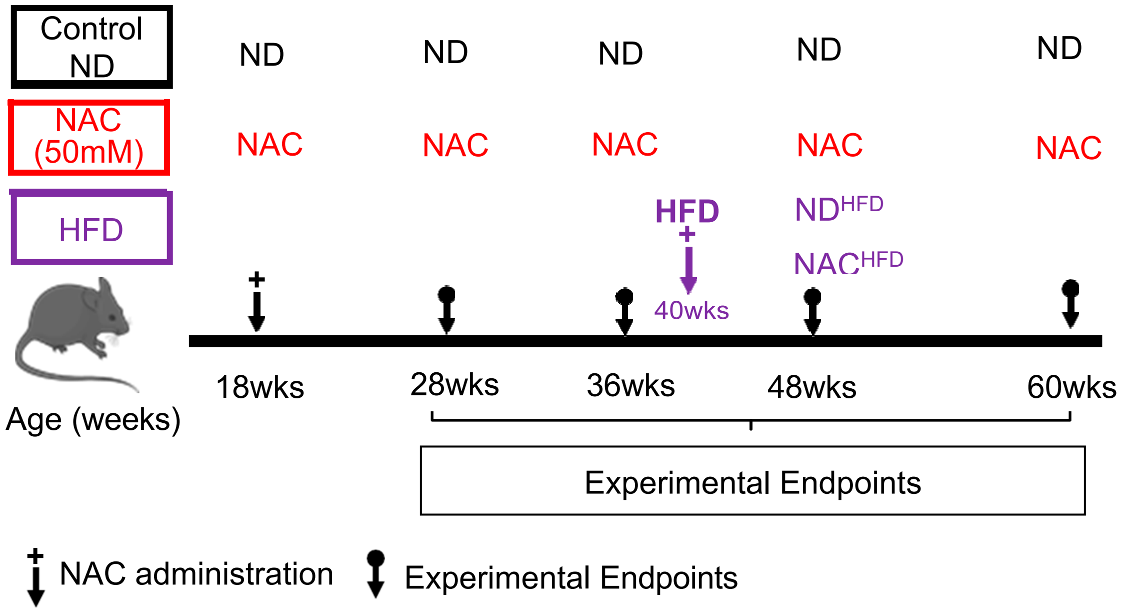

Supplemental Figure. S1: Experimental timeline for aging and HFD-challenge. Both normal diet (ND) and normal diet with NAC (NAC) experimental groups were established at 18 weeks of age. At 40 weeks of age, both ND and NAC mice were fed with HFD for 8 weeks to create ND^{HFD} and NAC^{HFD} experimental groups.

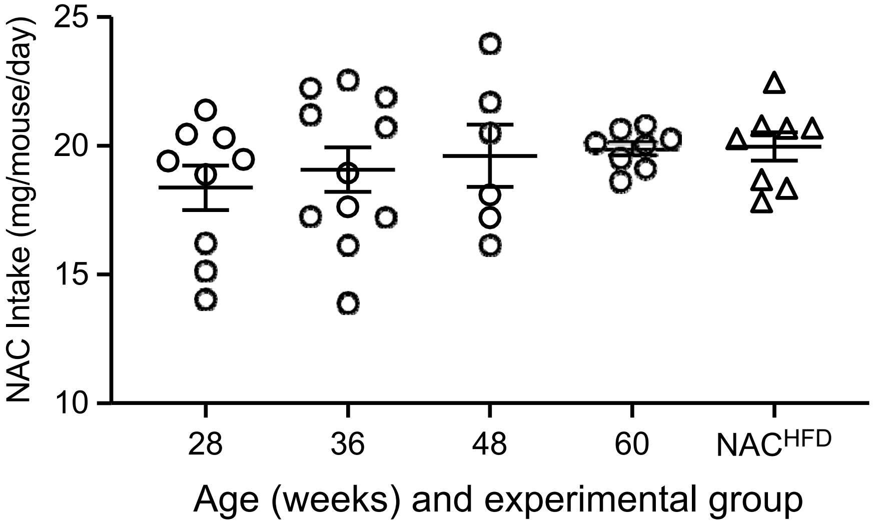

Supplemental Figure S2: NAC intake in experimental groups. 50mM NAC was administered in drinking water. NAC intake was measured at each timepoint and treatment condition over a 3-day period. Data are expressed as means ± SEM (n=6-11 mice per experimental group). There were no significant differences as determined using a one-way ANOVA.

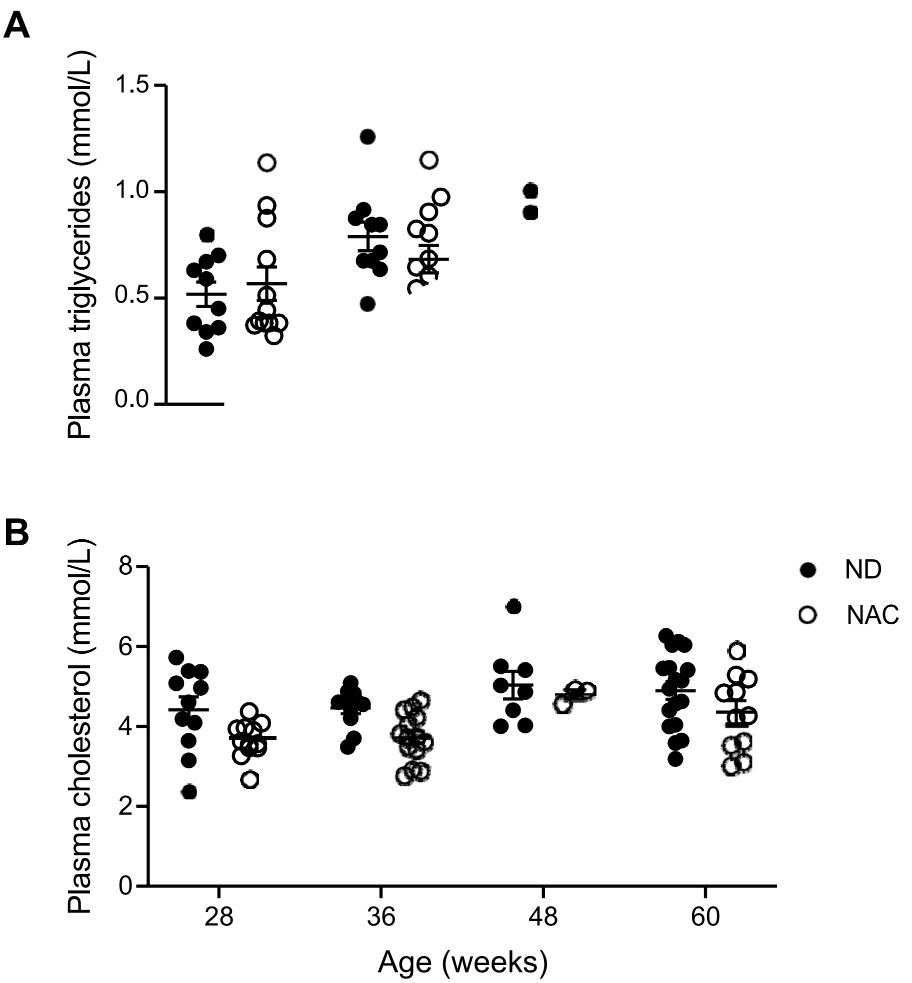

Supplemental Figure S3: Plasma triglycerides (**A**) and cholesterol (**B**) levels in normal diet (ND) and normal diet with NAC (NAC) experimental groups at 28, 36, 48 and 60 weeks of age. Data are expressed as means ± SEM (n=3-18 mice per experimental group).

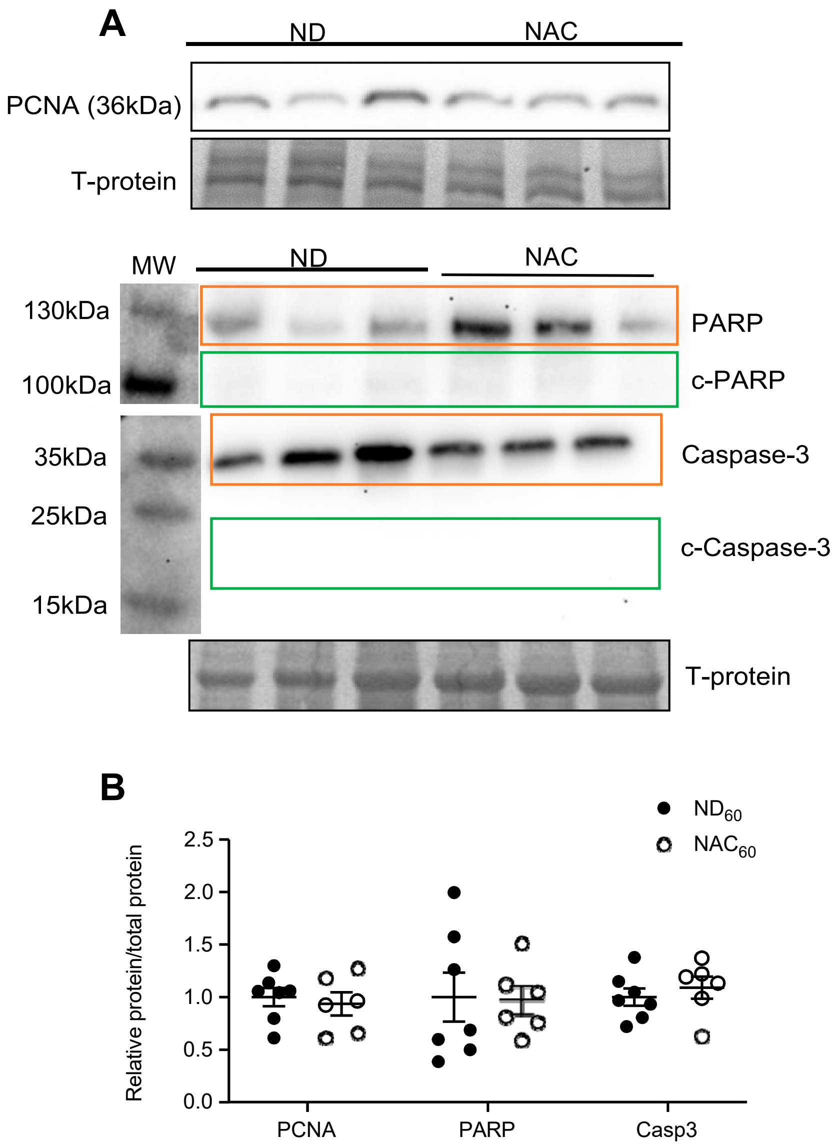

Supplemental Figure S4: (A) Representative western blot images and **(B)** densitometry quantification of cell proliferation (PCNA) and cell death markers (PARP and caspase 3) in isolated mouse islets at 60 weeks of age (n=6-7 mouse isolated islets/group). Data are expressed as means ± SEM.

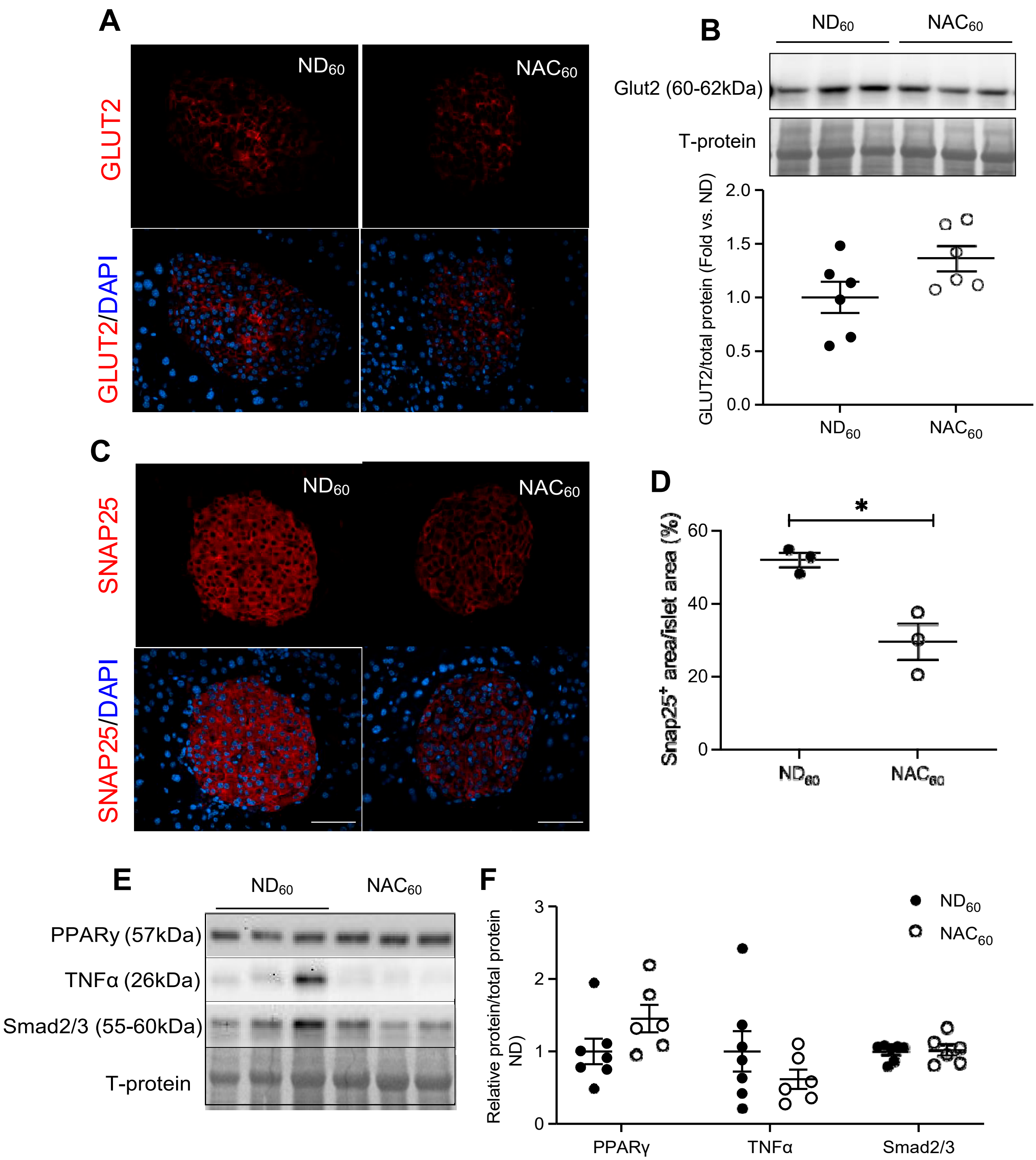

Supplemental Figure S5: (A) Representative images of immunofluorescence of GLUT2 (red) and **(B)** densitometry quantification of GLUT2. **(C)** Representative images of immunofluorescence of SNAP25 (red) and **(D)** quantification of SNAP25^{+} area to islet area. Nuclei are stained with DAPI (blue). Scale bars: 50μm. **(E)** Representative western blot images and **(F)** densitometry quantification of various proteins involved in inflammatory pathways: PPARγ, TNFα, and Smad2/3 in isolated mouse islets at 60 weeks of age (n=6-7 mouse isolated islets/group). Data are expressed as means ± SEM. *p<0.05; analyzed using unpaired Student's t-tests.

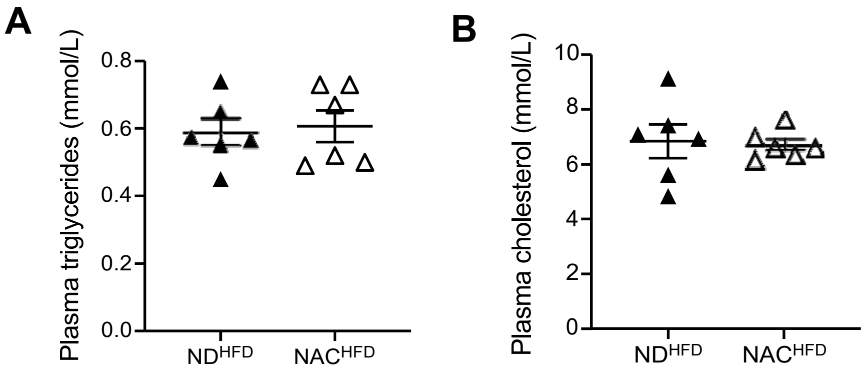

Supplemental Figure S6: Plasma lipid levels **(A)** Triglycerides and **(B)** cholesterol in HFD-challenged ND^{HFD} and NAC^{HFD} experimental groups 48 weeks of age. Data are expressed as means ± SEM (n=6 mice per experimental group). There were no significant differences as determined using an unpaired Student's t-test.
